# Poultices

**Published:** 1887-02-12

**Authors:** 


### Till the Doctor Comes.

[Inquiries and Communications for this column should be addressed, "Physician," care of the Editor of The Hospital.]

### X.—POULTICES.

*Linseed Poultice.*—Scald the basin to be used in its preparation, then put into it the proper quantity of linseed meal; add boiling water, and stir constantly with a wooden spoon or spatula till it is of the proper consistence; be careful not to add too much water. When mixed turn it out on a piece of clean rag or tow (muslin, called tiffany, about 2*d.* a yard, is better than rag or tow) of the requisite size, and spread it evenly over the surface. Next fold the sides of the muslin neatly into the margin of the poultice, and spread a little vaseline or sweet-oil over its surface. If this is done, and too much water has not been added, it will not adhere to the skin on removal, but; to prevent this, some persons prefer to lay a piece of very thin muslin over it. Apply it as warm as it can be borne; cover it with a thin sheet of Mackintosh, and apply a bandage, or, if on the body, pin a warm towel round the body to keep it in place. A poultice thus applied ought to keep hot for about four hours, and should then be removed.

*Charcoal Poultice.*—Made by adding two or more table-spoonfuls of finely-powdered charcoal to the linseed. It is most valuable in removing the smell from foul wounds.

*Opium Poultices.*—Pour a teaspoonful of laudanum over the surface of the linseed poultice. Used to relieve pain.

*Bread Poultice.*—Put the necessary quantity of stale bread into a basin, and pour over it sufficient boiling water to soak it thoroughly; let it remain for five minutes with a plate covering the basin, then drain off superfluous water, and place the bread between layers of muslin or soft old linen.

*Jacket Poultice.*—Two large linseed poultices, one to the chest, the other to the back, and kept in place by a towel pinned firmly round the body. Useful in many cases of acute lung disease.

*Mustard Plaster.*—Mix ground mustard with warm water to a thin paste, spread on brown paper or cloth; cover it with thin muslin, and apply it till surface is reddened; this ought to be on from twenty to thirty minutes. Mustard leaves are generally used now; they are much cleaner and more elegant, but give rise to more pain, and their effect is less.

*Yeast Poultices.*—Dry heat is often much more comfortable than moist, especially when poultices from their position, as on the face, cannot be everywhere closely applied to the skin. For this purpose bran poultices are the most comfortable ones. They are made by filling muslin bags with bran, and quilting them once or twice, after which they are heated in the oven, and applied. Two should be in use at the same time, one in the oven, the other on the patient. Chamomile flowers may be used instead of bran.

*Hot Sand-bags.*—In some cases of neuralgia and lumbago greater heat is required: then hot sand-bags will be found most serviceable. They also retain the heat a very long time, and by their weight exercise some pressure on the part, which again helps to relieve the pain. Sand-bags are made by filling with sand pieces of ticking sewn up as long and rather narrow bags; they must not be filled too full, or they cannot be so well adjusted to the part.

